# The EU-Joint Clinical Assessment Guidance Documents Fail to Address the Significance of Systematic Literature Reviews and Deviate from the State of the Art

**DOI:** 10.3390/jmahp14030037

**Published:** 2026-06-24

**Authors:** Beata Smela, Mondher Toumi, Samuel Aballéa, Steven Simoens, Laurent Boyer, Bruno Falissard, Renato Bernardini, Stefano Capri, Pascal Auquier

**Affiliations:** 1 Clever-Access, 30-415 Cracow, Poland; 2 Health Services Research and Quality of Life Center, Aix-Marseille University, 13385 Marseille, France; mto@inovintell.com (M.T.); laurent.boyer@univ-amu.fr (L.B.); pascal.auquier@univ-amu.fr (P.A.); 3 Inovintell, Mathenesserlaan 447B, 3023 GJ Rotterdam, The Netherlands; sab@inovintell.com; 4 Department of Pharmaceutical and Pharmacological Sciences, Catholic University of Leuven, 3000 Leuven, Belgium; steven.simoens@kuleuven.be; 5 Centre de Recherche en Epidemiologie et Santé des Populations, CESP, INSERM U1018, Université Paris-Saclay, 94800 Villejuif, France; bruno.falissard@gmail.com; 6 Department of Biomedical and Biotechnological Sciences, University of Catania, 95124 Catania, Italy; bernardi@unict.it; 7 School of Economics and Management, Cattaneo-LIUC University, 21053 Castellanza, Italy; scapri@liuc.it

**Keywords:** Joint Clinical Assessment, systematic literature review, Health Technology Assessment, PICO, EU-HTA, methodology

## Abstract

This paper summarizes an analysis of the Joint Clinical Assessment (JCA) subgroup’s recommendations for systematic literature reviews (SLRs). While the JCA offers clear guidance on study classification, exclusion criteria reporting, and PRISMA diagram use, several of its recommendations diverge from established best practices in evidence-based medicine (EBM). A comparison with recognized guidelines, such as those from Cochrane and EUnetHTA, reveals that the JCA guidance may lack reliability, comprehensiveness, and reproducibility. Aligning JCA recommendations with gold standards in SLR methodology would address these shortcomings and enhance methodological rigor.

## 1. Introduction

The EU Health Technology Assessment (HTA) regulation Ref was adopted on 15 December 2021, came into force on 11 January 2022, and applied from 12 January 2025. This EU-HTA regulation establishes a support framework and procedures for cooperation of member states on health technology at the EU level. It creates an EU HTA Member States Coordination Group with two main goals: conducting Joint Clinical Assessments (JCA) and offering Joint Scientific Consultations (JSC) on medicinal products and medical devices.

The JCA aims to assess the level of certainty of the comparative effectiveness of medicinal products. The framework PICO (Population, Intervention, Comparator, Outcome) is the pivotal tool to define the evidence requirements for clinical evidence assessment.

While the systematic literature review (SLR) will be central to the JCA, little attention has been given to this topic, and no guidance has been developed for SLRs. A recent study focused on simulating the evidence generation requirements under the JCA predicted that between 8 and 31 Population, Intervention, Comparator, Outcome (PICO) frameworks would be necessary for two anticancer investigational medicinal products, depending on the treatment landscape and the specific requirements of each member state. This indicates that health technology developers (HTDs) may be required to submit up to 31 distinct comparative effectiveness assessments [1]. The JCA subgroup recently published a PICO exercise for two oncology products, identifying 13 PICOs for each [2].

At the time of submission of the JCA dossier, a limited number of comparative clinical trials will be available, typically including one phase II and one phase III trial, or a single phase II/III trial, or just a phase II trial, and even non-comparative single-arm trials. When comparing these trials, they are often using a placebo as the reference Comparator. To address the multiplicity of PICOs and generate comparative effectiveness, direct evidence comparisons will be insufficient and may only inform a minority of PICOs. The majority of PICOs will require systematic literature reviews (SLRs) followed by indirect treatment comparisons (ITCs). The quality of the SLRs will be crucial for the comparative effectiveness assessment. Both SLR and ITC will play a vital role in documenting the comparative effectiveness for EU-JCA purposes.

While SLRs are critical to assess JCA comparative effectiveness, it is unclear why a specific guidance document was not developed to address methodological recommendations for conducting SLRs. Moreover, the JCA subgroup does not recommend a specific guideline for conducting SLRs, but emphasizes that the submission dossier should adhere to the principles of evidence-based medicine (EBM) with no further specifications [3,4]. This remains an open, vague, non-actionable recommendation. Nevertheless, through JCA guidance documents and reporting templates, several recommendations are offered that outline the expectations of the JCA subgroup regarding SLR generation.

While the JCA subgroup offers well- established recommendations for studies classification/hierarchy, reporting of reasons for exclusion, and PRISMA diagram generation, several requirements or recommendations are not aligned with the state of the art.

This manuscript discusses the requirements for SLR development for JCA that deviate from the state of the art.

## 2. Scope of Literature Search

### 2.1. No Requirement for Embase

The guidance on filling in the JCA dossier template for medicinal products specifies: “The search in bibliographic databases at least is to be conducted in MEDLINE (inclusive “in-process & other non-indexed citations”) and the “Cochrane Central Registry of Controlled Trials” database” [5].

The guidance on filling in the JCA dossier template for medicinal products specifies that the search in bibliographic databases, ‘at least is to be conducted in MEDLINE… and the Cochrane Central Registry of Controlled Trials’, and that ‘in addition, a search can be conducted in further databases (e.g., Embase, CINAHL, PsycINFO, etc.).’ By making Embase optional rather than mandatory, the guidance permits dossiers in which Embase is omitted, even though such an omission would not meet the methodological standards of any reference HTA agency or the Cochrane Handbook. The absence of Embase from the mandatory list, irrespective of whether the guidance also articulates a general principle of completeness, is the methodological flaw we identify, because operational guidance documents are read, applied, and audited for what they require, not for the general aspirations they invoke.

This is not aligned with the state of the art according to the Cochrane Handbook, which advises that, at a minimum, systematic reviews should search MEDLINE and Embase, along with Cochrane Central Register of Controlled Trials (CENTRAL) [6].

Embase includes all items indexed in MEDLINE, along with an additional 3000 journals (over 7 million records), featuring a significant number of European and non-English publications [7,8,9]. Some analyses have demonstrated that Embase provides more comprehensive coverage of controlled clinical trials compared to MEDLINE. For instance, Embase indexes 16% more studies on conditions such as rheumatoid arthritis, low back pain, and osteoporosis [7]. Its broader scope also extends to research on pharmacological treatments and drug-related adverse events [7]. Moreover, Embase includes around 5 million conference abstracts from around 16,000 conferences, which helps reduce publication bias. The database frequently offers broader access to European and international clinical practice guidelines, which play a key role in preparing global regulatory dossiers and documentation for multinational clinical trials [8,10].

For European-focused projects—regulatory, epidemiological, or clinical—Embase is the better choice. The two databases are complementary, and for comprehensive coverage, especially for HTA or evidence synthesis projects, it is still standard practice to search both [7,9,10].

Not including Embase can lead to missing relevant studies and potentially bias the review’s conclusions. Ensuring that all relevant data sources are comprehensively covered is critical to maintaining the methodological rigor expected from high-quality SLRs, particularly in the context of JCAs. Currently, the JCA does not meet the minimum requirements established by member states HTA bodies, even though it is expected to inform these entities.

### 2.2. Excluding Abstracts

JCA report template specifies that studies must have enough documentation to assess their methods and results, and therefore recommends excluding studies with only abstracts or posters available, when setting inclusion and exclusion criteria [5].

Including conference abstracts is crucial for reducing publication bias, ensuring that the most recent evidence is considered, and guaranteeing comprehensive EBM practice. However, it is important to acknowledge that abstracts may sometimes be incomplete or partial; nonetheless, they may also provide additional useful information about complementary analyses or represent the first publication of a study and thus should be considered carefully. Excluding them may result in missing relevant studies and biasing the review’s conclusions. Exhaustiveness of evidence is fundamental to EBM [6,11,12].

Omitting abstracts also diverges from the recommendations set forth by EUnetHTA [13,14]. According to EUnetHTA guidance, only around half of all studies initially presented as abstracts are eventually published in full, and abstracts are more likely to be published as full articles if they demonstrate a favorable treatment outcome or statistically significant findings [13,14,15]. Conference abstracts typically offer minimal information regarding study design and often report limited outcome data [16]. EUnetHTA advises against routinely including abstracts in evidence searches and recommends that reviewers should make every effort to obtain the complete study report or additional study details before deciding whether to incorporate the findings into the assessment [13,14,16]. Nevertheless, in situations where systematic searches of published literature result in few or no relevant records—or when existing data is inconsistent—searching conference abstracts and proceedings may be justified to uncover supplementary studies [13,14,16]. These abstracts and proceedings can be located through bibliographic databases that catalog conference materials [17], such as Embase, BIOSIS Previews, and Scopus, as well as through manual searches of conference booklets, journal supplements, and event websites [16].

So even if EUnetHTA is more nuanced, it does not exclude abstracts systematically from the search.

Excluding abstract and Embase from the sources of SLR goes against the widely accepted standards in evidence-based synthesis and medicine. It should be reconsidered.

## 3. Using the Risk of Bias Version 1 Tool (RoB 1) for Clinical Trial Evidence Quality Assessment

The JCA guidance recommends using the RoB 1 instrument to assess risk of bias, though the updated risk of bias version 2 (RoB 2) tool was developed in 2019 to replace it [18]. RoB 2 addresses previous limitations and improves the standardization, transparency, and reproducibility of risk of bias assessments in randomized controlled trials (RCTs) [6,19].

Compared to RoB 1, which assesses six broad areas of bias, RoB 2 streamlines the evaluation into five specific domains and removes the “other bias” category. RoB 2 introduces an overall risk of bias judgment and incorporates structured signaling questions to guide reviewers toward more consistent and transparent assessments. These signaling questions allow for more nuanced judgments (“low risk,” “some concerns,” or “high risk”), improving the precision and reproducibility of evaluations compared to the original RoB 1 tool [6,20].

RoB 1 offers a general evaluation of each study considering all outcomes, whereas RoB 2 performs a result-level assessment for specific outcomes. RoB 2 enables reviewers to evaluate context and endpoints with greater accuracy, thereby enhancing the precision of their judgments compared to RoB 1 [6].

RoB 2, compared to RoB 1, has several advantages. It offers clearer guidance with structured signaling questions and focuses on result-level assessments, enhancing relevance for SLRs. Transparency is improved by removing ambiguous categories such as “other bias” and “unclear risk”. Interestingly, the analysis conducted by Babic et al. showed that, among 768 analyzed Cochrane reviews, 78% (602 reviews) contained domain ‘other bias’ in the risk of bias tool, covering 7811 RCTs [21]. The authors observed a wide variety of sources categorized as ‘other bias’ in the RoB tool applied by researchers, along with variability in the interpretation of identical supporting justifications. Such inconsistency in evaluating the risk of other bias with RoB 1 undermines the reliability and comparability of SLRs’ findings [21].

The JCA recommends RoB 1, primarily due to its shorter operation time compared to the upgraded version [5,18]. This recommendation raises two important questions. Firstly, in terms of the quality of reports focusing solely on clinical evidence, should assessors and co-assessors not employ the highest quality tool endorsed by a reputable institution like Cochrane? Is the difference in operating time significant enough to consider using a second-best tool that was replaced by a more effective one?

Numerous factors influence the time required to utilize RoB 2, with experience being a significant one. While added complexity is generally linked to increased duration, one outlier publication indicates that mean assessment times are similar for both tools [20]. This decision to use RoB 1 warrants further consideration, including potentially piloting RoB 2 with trained reviewers. The guidance document acknowledges the limitation of RoB 1 and the value of RoB 2 indirectly by specifying: “It is nevertheless advisable that assessors familiarise themselves with RoB 2, so they can pay attention to more subtle mechanisms of bias and describe these in a JCA, if required” [18]. It raises the question of why RoB2 is not used directly while providing adequate training to assessors.

The JCA reporting template requests RoB assessments for each outcome, which contradicts the purpose of the RoB 1 tool, designed for study-level bias assessment [18]. Although technically possible, using RoB1 for outcome-level assessments is not recommended by Cochrane. It lacks the structure for reliable outcome-level evaluations and can lead to inconsistencies and subjectivity. Cochrane discourages routine outcome-level use of RoB1 due to its limited suitability for specific issues like differential blinding or missing data rates [6].

Utilizing RoB2 in the JCA dossier will help ensure that RoB evaluations adhere to contemporary standards and are uniformly applied across various outcomes.

Using RoB 1 goes against the widely accepted standards in evidence synthesis and EBM. It should be reconsidered.

Although the recommendation to use RoB concerns the clinical validity of submitted studies, it also applies to indirect treatment comparisons and underlying clinical studies evidence, which form the basis for most PICOs assessments.

## 4. Quality Assessment of Evidence Was Overlooked

Although RoB assesses clinical trial evidence quality, it is not enough to evaluate the overall quality of a body of evidence. To do so, researchers need to use a specific tool to determine quality for eligibility for SLR.

Tools such as Grading Recommendation assessment, development, and evaluation (GRADE) specifically assess the certainty of the body of evidence, measuring our confidence in the overall findings based on all available evidence, and focusing on the final outcomes [6]. For example Cochrane [6], National Institute for Health and Care Excellence (NICE) [22], World Health Organization (WHO) [23], and the Centers for Disease Control and Prevention (CDC) [24] use GRADE. The absence of an endorsed tool can result in inconsistency in the assessment of clinical trial evidence among different JCAs or even within the same JCA for different PICOs’ related evidence. Without a standardized and widely accepted framework, assessors may employ diverse methodologies, potentially leading to discrepancies in the evaluation of evidence quality. Such inconsistencies could compromise the reliability and reproducibility of JCA reports, which are critical when these evaluations are utilized to guide policy decisions and healthcare strategies at the European level.

Global health organizations, such as NICE and Cochrane, have developed clear methodologies for assessing evidence quality [6,22]. The JCA’s decision not to recommend a standardized tool like GRADE may result in misalignment with these best practices. Adopting such tools would align JCA with international standards and enhance the credibility and acceptance of its findings.

It is therefore recommended to use GRADE after assessing individual studies (with RoB tools), as it enables judging how trustworthy and reliable the pooled body of evidence is for decision-making [6].

Not recommending a tool for clinical trial reporting quality assessment also goes against the widely accepted standards in evidence synthesis and EBM. It should be reconsidered.

## 5. Time Pressure Will Affect Quality

HTD has 90 days to develop the JCA dossier [4,5]. Based on this recommendation, the fact that the dossier should be submitted 45 days prior to the Committee for Medicinal Products for Human Use (CHMP) opinion, and considering the median duration of the first regulatory stop of the clock, the conclusion can be made that for half of the dossiers, the time for development of the dossier will be less than 90 days [25]. Defining the PICO scope takes up to 100 days, which may be reduced to 90 days. Developing a submission dossier within 90 days is technically impossible. Therefore, HTD must anticipate the scope and prepare the dossier in advance. When the final scope is available, HTD will need to adjust its dossier accordingly.

Guidelines advise that groups conducting SLRs should involve individuals with subject-matter expertise relevant to the area under investigation, alongside specialists in SLR’s methods and statistical analysis. The structure of the review team, the possibility to exchange doubts between team members, and time for consensus can notably influence the quality, credibility, and outcomes of the review. Ensuring openness and clarity regarding team composition is essential to minimize the risk of bias and maintain methodological integrity [6,26]. To form such a team and facilitate information exchange between team members, adequate time is required.

This approach poses challenges when considering the multiple PICOs and submission timelines for JCA dossiers.

The 3-month timeline for evidence to be considered up to date is too short compared to, for example, NICE’s 6-month window [27]. Not knowing the PICOs in advance exacerbates this problem. Performing updates of searches, extractions, quality controls, and ITC updates should be timely. Last-minute report updates can be disruptive.

Similarly, EUnetHTA advises that information specialists should be an essential component of the SLR or HTA team from the project’s outset. Search strategies ought to be subjected to peer evaluation to guarantee their robustness and quality. The search activities should be documented contemporaneously and communicated in a clear and transparent way [13].

The current approach proposed for JCA submission and reporting disrupts time management and raises the risk of errors due to time pressure and the complexity of updating SLRs when PICOs are revised. Handling multiple PICOs under time pressure demands significant resources, which does not comply with best practices.

Not providing a reasonable time to conduct SLR goes against the widely accepted standards in evidence synthesis and EBM. It should be reconsidered.

## 6. Other HTA Agencies’ SLRs’ Requirements and EUnetHTA Recommendations on SLR

HTA bodies worldwide, at both national and regional levels, offer guidance on healthcare interventions that may be eligible for public funding or reimbursement. These entities base their decisions on comprehensive dossiers submitted by manufacturers, with each organization providing its own specific templates or criteria to support the preparation of required materials. Evidence from clinical studies—particularly RCTs—forms the cornerstone of economic analyses, which in turn enables HTA institutions to deliver decisions grounded in scientific evidence. Across the reviewed agencies from various global regions (see Table 1), there is a shared understanding that clinical evidence must be gathered in a systematic way, from multiple sources, based on a predefined PICO, and that the quality of the collected data must be critically appraised to ensure maximum bias reduction. Guidelines from HTA agencies and EBM organizations have been taken into account by EUnetHTA and reflected in detailed recommendations published in 2015 [14] and 2019 [13], outlining the specific steps for conducting SLRs, which can then serve as a solid foundation for making health-related decisions in an international context. The recommendations and templates for JCA dossier submission and report do not align with EUnetHTA’s oldest SLR guidelines. It is recommended that JCA adopt Cochrane guidelines to ensure state-of-the-art EBM standards.

## 7. Conclusions

It is to be expected that the majority of PICOs will be addressed through SLR, followed by ITC when feasible, while direct evidence will only document a low number of PICOs. However, guidance documents disproportionately address direct comparison. SLR is central to most PICOs and merits dedicated guidance. While no specific guidance for SLR has been developed, the topic is mentioned in various places. Adhering to available EUnetHTA guidelines for SLR would have provided clear, concise guidance aligned with the state of the art [13,14]. This manuscript summarizes the essential requirements for performing SLR for JCA submission, while SLR on performing SLR was obviously not conducted; as a consequence, recommendations deviate from best practices as outlined by Cochrane and EUnetHTA guidelines. For example, when EUnetHTA developed its guidelines for conducting SLR, they reflected on the state-of-the-art references available at that time [6,17,37,38,39,40,41]. Guidance documents are intended to reflect the state of the art in EBM as specified in the EU-HTA Regulation [3], but the recommendations in JCA documents deviate significantly from this standard. This may expose JCA to issues such as unreliability, errors, lack of comprehensiveness, and lack of reproducibility. Adhering to the Cochrane guideline, regarded as the highest standard in the field for SLR, is a simple way to address these concerns and avoid inappropriate recommendations. Recommending the EUnetHTA SLR guidelines would also be advisable, as they have been recognized for their high quality.

## Figures and Tables

**Table 1:** Requirements for the SLR process.

Agency	SLR Needed	The Necessity of Defining PICOS Criteria	Databases	Other Specified Sources	Selection Process	PRISMA Required	Critical Appraisal
NICE [28]	Yes	Yes	Medline, Embase, and Cochrane	Unpublished data, reference searching, citation searching, inclusion list of systematic reviews, websites	Double-screening done by independent reviewers	Yes	Validated tools specific to the study design and use case
HAS [29]	Yes	HAS recommends following the Cochrane Handbook	HAS recommends following the Cochrane Handbook	Relevant websites (government agencies, learned societies, conferences), legislative and regulatory texts	HAS recommends following the Cochrane Handbook	Yes	HAS recommends following the Cochrane Handbook
IQWIG [30]	Yes	Yes	Medline, Embase, Cochrane	Trial registries, manufacturer data, HTA agency websites, PROSPERO, Dynamed, UpToDate	Double-screening done by independent reviewers	Yes	Defined by the basic principles of good clinical practice
CADTH [31,32,33]	Yes	Yes	Medline, Cochrane	Trial registries, websites of INAHTA agencies, manufacturers’ websites, internet search tools, consultation with experts and agencies	No information	No information	Not reported
PBAC [34]	Yes	Yes	Medline, Embase, Cochrane	Trial registries, reference lists, marketing approval dossiers, company databases	No information	Yes	Cochrane RoB 2
EUnetHTA [13,14]	Yes	Yes	Medline, Embase, Cochrane	SLR should regularly include a search for unpublished literature to identify both unpublished studies and unpublished data from published studies	Double-screening done by independent reviewers	Yes	Use the RoB concept of the Cochrane Collaboration to assess the internal validity of RCTs (guideline on internal validity of RCTs published in 2015 [14], therefore, could not contain information on RoB 2)
JCA [5,18,35,36]	Yes	Yes	Medline, Cochrane	Trial registries, manufacturer data	No detailed information	Yes	Use of the RoB1; however, mandatory completion of signaling questions specific to RoB2 leads to methodological inconsistency and a lack of clarity

## Data Availability

No new data were created or analyzed in this study.

## Abbreviations

The following abbreviations are used in this manuscript:

Abbreviation	Full Form
CADTH	Canadian Agency for Drugs and Technologies in Health
CDC	Centers for Disease Control and Prevention
CENTRAL	Cochrane Central Register of Controlled Trials
CHMP	Committee for Medicinal Products for Human Use
EBM	Evidence-Based Medicine
EU-HTA	European Health Technology Assessment
EUnetHTA	European Network for Health Technology Assessment
GRADE	Grading of Recommendations Assessment, Development and Evaluation
HAS	Haute Autorité de Santé
HTDs	Health Technology Developers
IQWIG	Institute for Quality and Efficiency in Health Care
ITCs	Indirect Treatment Comparisons
JCA	Joint Clinical Assessment
JSC	Joint Scientific Consultations
NICE	National Institute for Health and Care Excellence
PBAC	Pharmaceutical Benefits Advisory Committee
PICO	Population, Intervention, Comparator, Outcome
PRISMA	Preferred Reporting Items for Systematic Reviews and Meta-Analyses
RoB 1	Risk of Bias version 1 tool
RoB 2	Risk of Bias version 2 tool
SLRs	Systematic Literature Reviews
WHO	World Health Organization
